# Supplementary material for: Wetter climates select for higher immune gene diversity in resident, but not migratory, songbirds
Source: Proc Biol Sci. 2020 Jan 29;287(1919):20192675. doi: 10.1098/rspb.2019.2675 (PMC7015325; doi:10.1098/rspb.2019.2675)
Supplement: Supporting Information [file rspb20192675supp1.pdf]

Appendix S1 Designation of species ranges and climate data extraction

Information on the distribution of the species in the current study was obtained from distribution maps provided as shapefiles by Birdlife International and Handbook of the Birds of the World (http://www.birdlife.org/datazone; accessed April 2017). We used the category 'Season' within the shapefiles to select the appropriate distribution for each species. 'Season' had five possible options: 1 = resident, 2 = breeding season, 3 = non-breeding season, 4 = passage, 5 = seasonal occurrence uncertain. We excluded distributions categorised as 4 or 5. In the case of species with a global distribution, such as *Hirundo rustica*, we used only the African and Palearctic distributions. The current study focused on species which could be classed as being year-round residents (Season = 1) in either the Palearctic or sub-Saharan Africa, as well as species which perform long-distance migration between these two regions (two distributions: Season = 2 and 3). There were some species in our study that perform short-distance migration within the Palearctic, such as *Anthus pratensis*, *Chloris chloris* and *Erithacus rubecula*. For the purposes of this study, these species were treated as Palearctic residents and the distributions for Seasons 1, 2 and 3 were combined to extract the climate data.

Climate data for the distribution range of each species was extracted from the CRU database using the 'extract' function in the R package 'raster' (1). Median values for temperature and precipitation were obtained for each month from January 1901 up to and including December 2017 (Table S2). This data was used to summarise the median, minimum and maximum temperature and precipitation for each year and then across years, resulting in a single median, minimum and maximum value for temperature and precipitation for each species.

Appendix S2 Categorising the habitat type of species

We characterised the general habitats of species using information collected from the habitat descriptions on the Handbook of Birds of the World Alive website (Table S1: data collected on Aug 21st to 24th 2018). Species were categorised as living close to water if any of the following terms were included: inhabits swamps, marshes, mangroves, wetlands, riverine forests or if they were described as living near water generally. If water was not mentioned in the habitat description they were not classed as living close to water. We categorised species as living in lowland regions if they were described as a lowland species or the maximum recorded altitude for the species was ≤ 2000 m, otherwise they were not classed as lowland species. In line with previous findings, the migrants in our study occupy similar habitat-types in both their breeding and wintering ranges (2). These species were therefore given a single habitat classification.

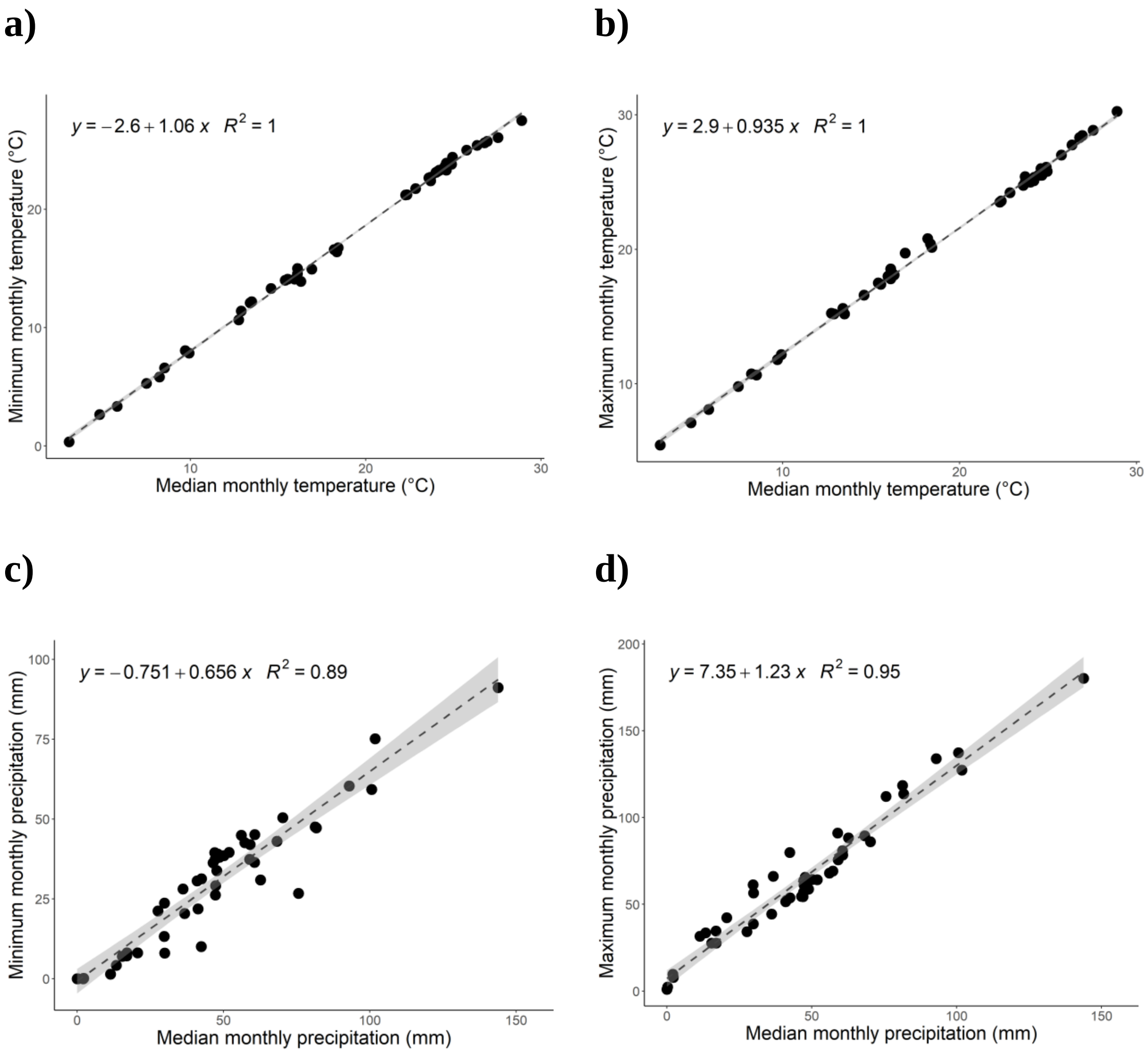

Figure S2 Relationships between the median and minimum/maximum values for temperature and precipitation. The precipitation and temperature data are based on monthly records from 1901 to 2017 collated by the Climatic Research Unit at the University of East Anglia. **a)** Relationship between median and minimum monthly temperatures. **b)** Relationship between median and maximum monthly temperatures. **c)** Relationship between median and minimum monthly precipitation. **d)** Relationship between median and maximum monthly precipitation. Dashed line and shaded areas show linear regressions with 95% confidence intervals. $n_{species} = 37$.

Appendix S3 Estimating demographic effects on MHC-I diversity

We performed three separate analyses to assess whether demographic processes influenced our estimates of MHC-I diversity. First, we measured 'haplotype redundancy' for each species. The term haplotype redundancy refers to more than one allele, at the level of the nucleotide sequence, coding for the same amino acid sequence. Lower haplotype redundancy is expected in smaller populations where the effects of genetic drift are more pronounced (3). Haplotype redundancy was calculated by dividing the number of unique alleles at the nucleotide sequence level by the number of alleles at the amino acid sequence level for each individual. Secondly, we measured the synonymous substitution rate (dS) across the non-PBR sites of MHC-I alleles for each species. Under the expectations of neutral theory, dS reflects the mutation rate and thereby neutral genetic variation, which is expected to be higher in species with a more stable demographic history leading to larger effective population sizes (3,4). dS across the non-PBR sites of MHC-I for each species was calculated using the Nei-Gojobori model in Molecular Evolutionary Genetics Analysis (MEGA) version 6 (5). We have previously used haplotype redundancy and dS to estimate the effect of demographic processes on MHC-I diversity and further details of these approaches can be found in O'Connor *et al.* (6). Thirdly, we used range size (total area in meters) as an approximation of the population size of each species (7–9). In addition to its effect on population size, range size has also been shown to be positively correlated with the species richness of pathogens, making it in itself a potentially confounding factor, especially if species with larger ranges differ in the climatic conditions they experience compared to species with smaller ranges (10).

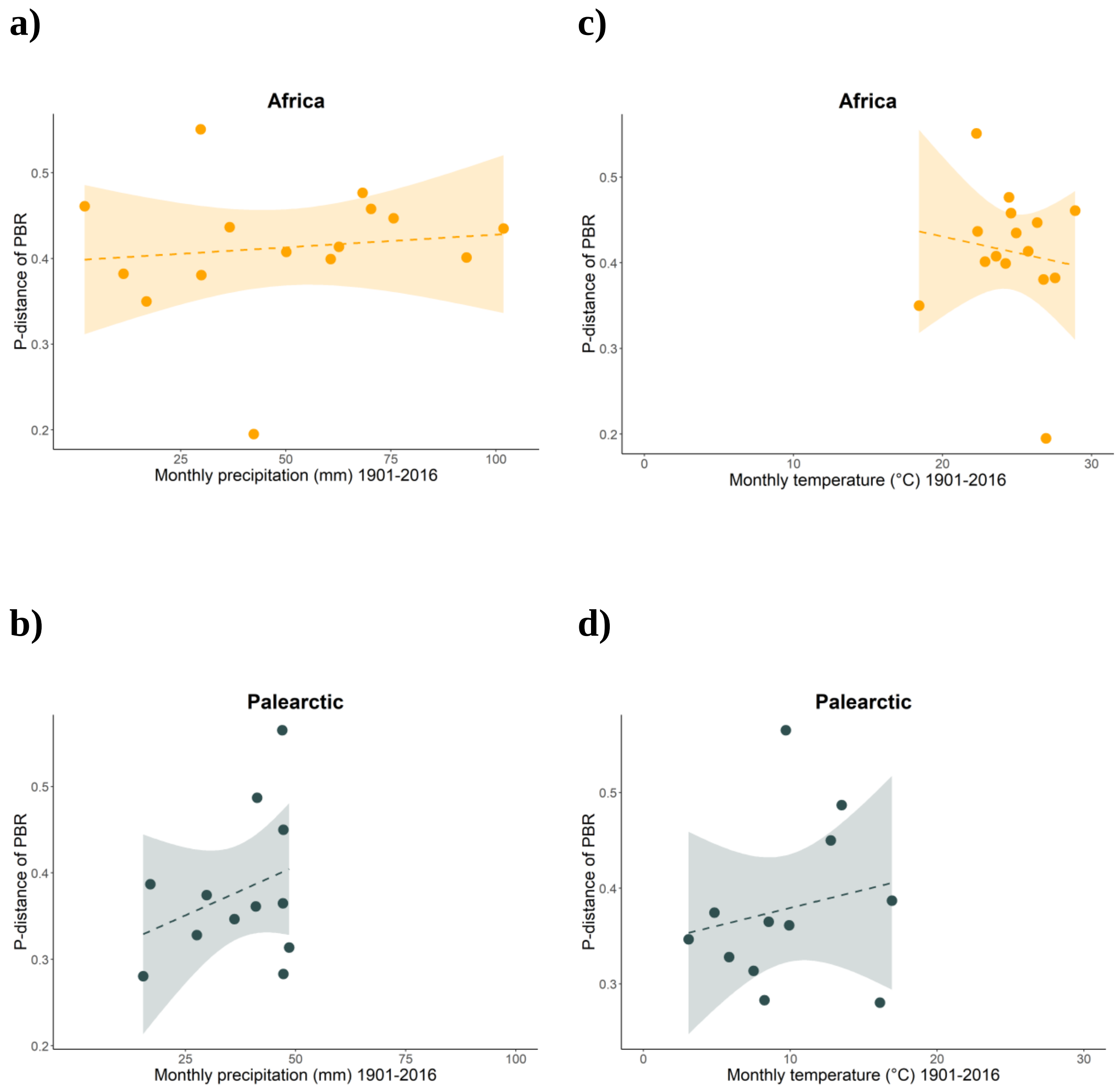

Figure S3 Relationship between climatic factors and pairwise sequence divergence (P-distance) in the peptide binding region of MHC-I alleles in African and Palearctic resident species ($n_{species}$ = 27). Relationship between median monthly precipitation and the mean P-distance of MHC-I alleles per individual in African residents **(a)** and Palearctic residents **(b)**. Relationship between median monthly temperature and the mean P-distance of MHC-I alleles per individual in African residents **(c)** and Palearctic residents (**d**). Dashed lines and shaded areas show linear regressions with 95% confidence intervals.

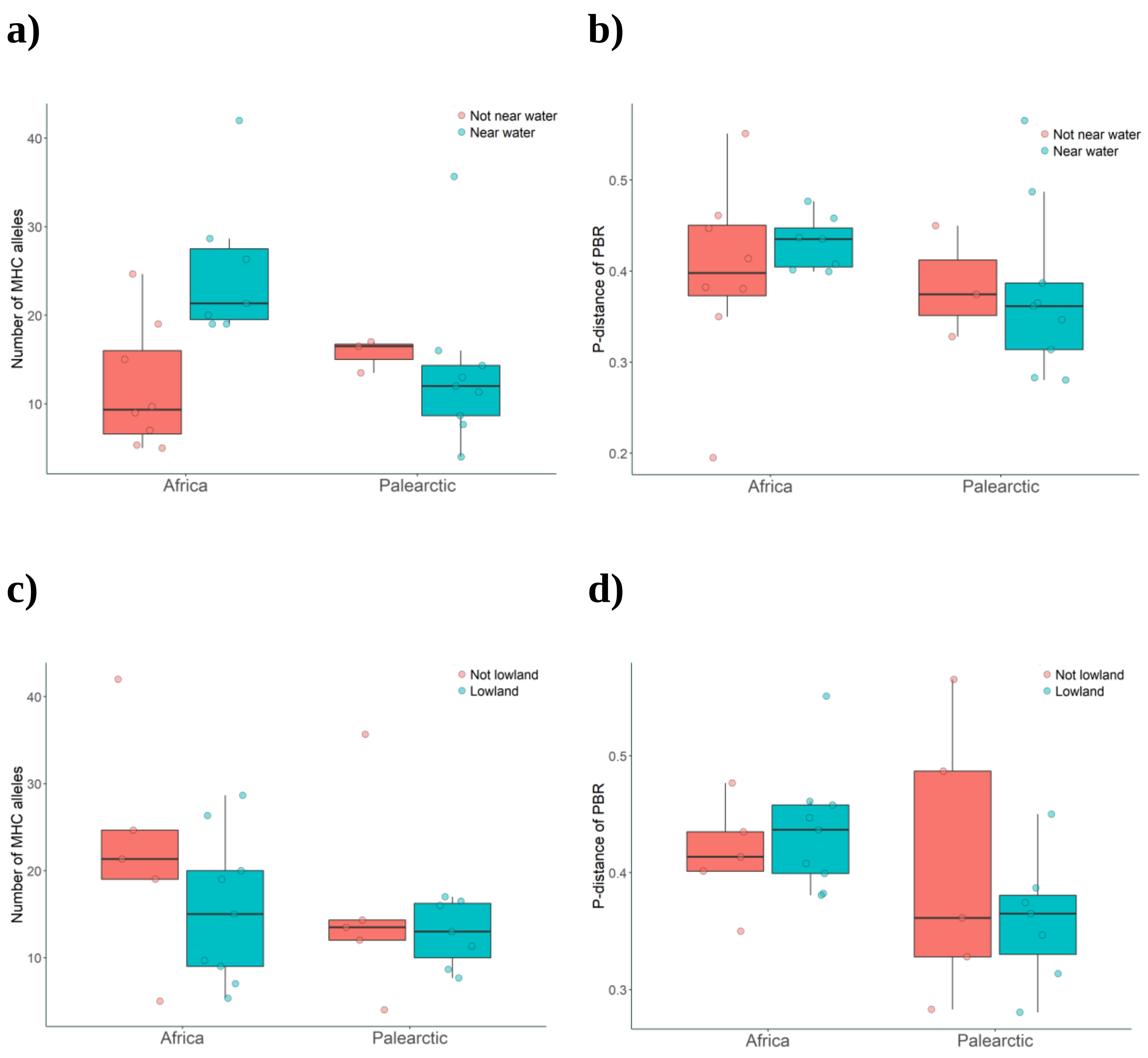

Figure S4 Relationship between MHC-I diversity and the habitat characteristics of the ranges of the resident species ($n_{species}$ = 27)**.** Relationship between living close to water or not in African and Palearctic resident species and the mean number of MHC-I alleles per individual (**a**) or mean sequence divergence (P-distance) in the peptide-binding region (PBR) of MHC-I alleles (**b**). Relationship between living in lowland habitats or not for African and Palearctic resident species and the mean number of MHC-I alleles per individual (**c**) or mean sequence divergence (P-distance) in the peptide-binding region (PBR) of MHC-I alleles (**d**). Data shown in box-plots (central line depicts the median, the lower and upper hinges correspond to the first and third quartiles and whiskers extend to

the highest value within 1.5 * the interquartile range) overlaid with the individual data points for each species.

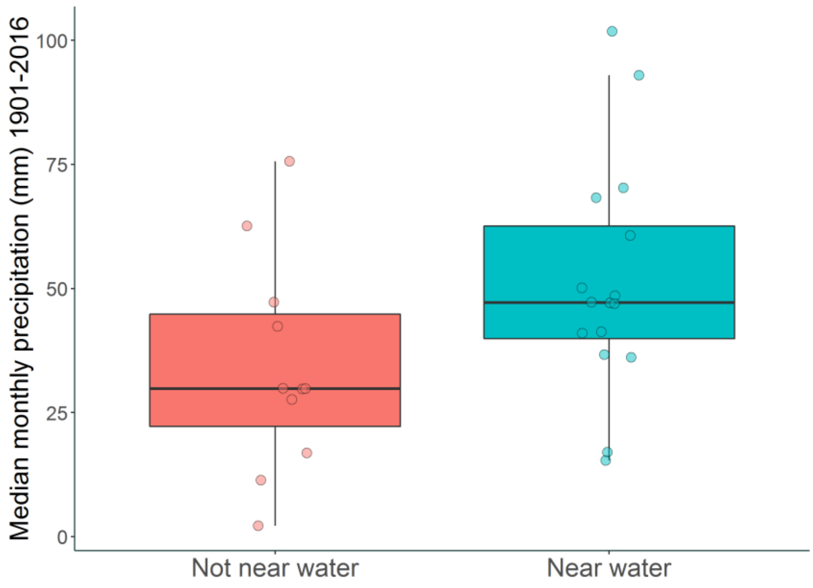

Figure S5 Difference in the median monthly precipitation in the distribution ranges of resident species ($n_{species}$ = 27) either classed as living close to water or not. Shown in box-plots (central line depicts the median, the lower and upper hinges correspond to the first and third quartiles and whiskers extend to the highest value within 1.5 * the interquartile range) overlaid with the individual data points for each species.

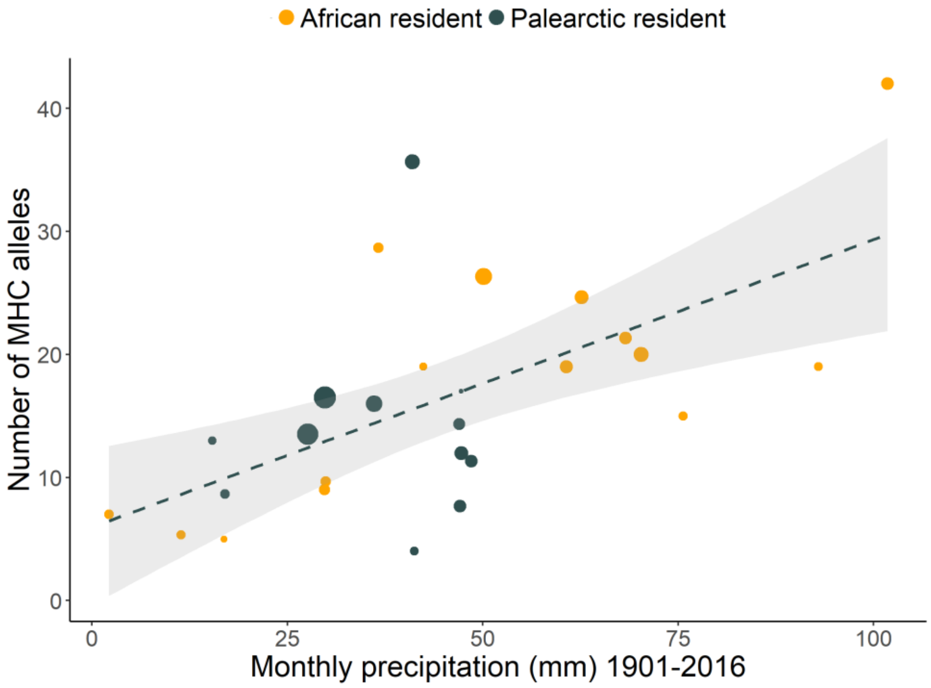

Figure S6 Relationship between median monthly precipitation and the mean number of MHC-I alleles per individual in African and Palearctic resident species accounting for range size ($n_{species}$ = 27). The size of the points are proportional to the total area of the distribution range of each species. Dashed line and shaded area shows linear regressions with 95% confidence intervals.

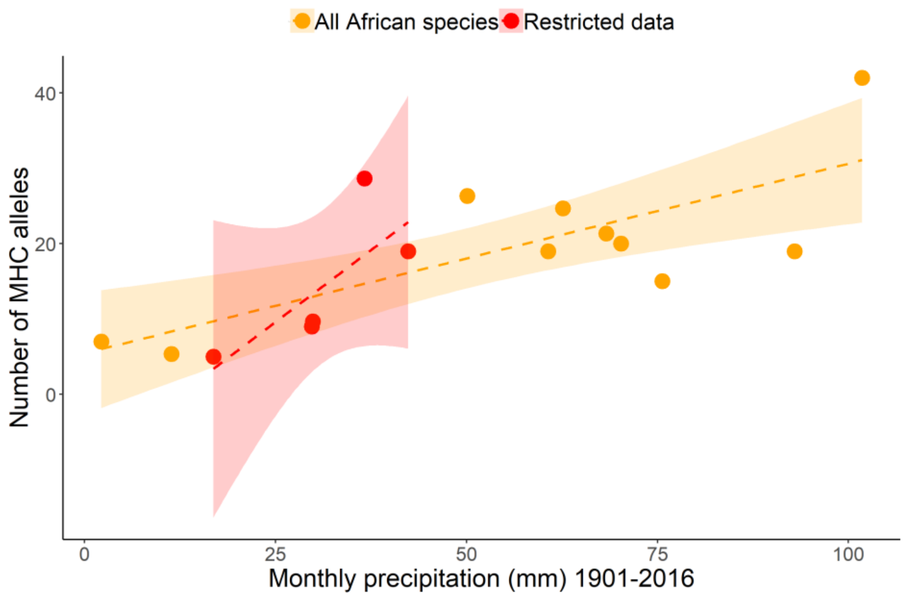

Figure S7 Comparison of the relationship between the number of MHC-I alleles in African resident species depending on the range of precipitation examined. Red dots show the African species which experience median monthly precipitation within the same range as Palearctic resident species (15.4 to 48.5 mm per month, $n_{species} = 5$). Orange dots show all the African resident species in the dataset ($n_{species} = 15$). Dashed lines and shaded areas show linear regressions with 95% confidence intervals.

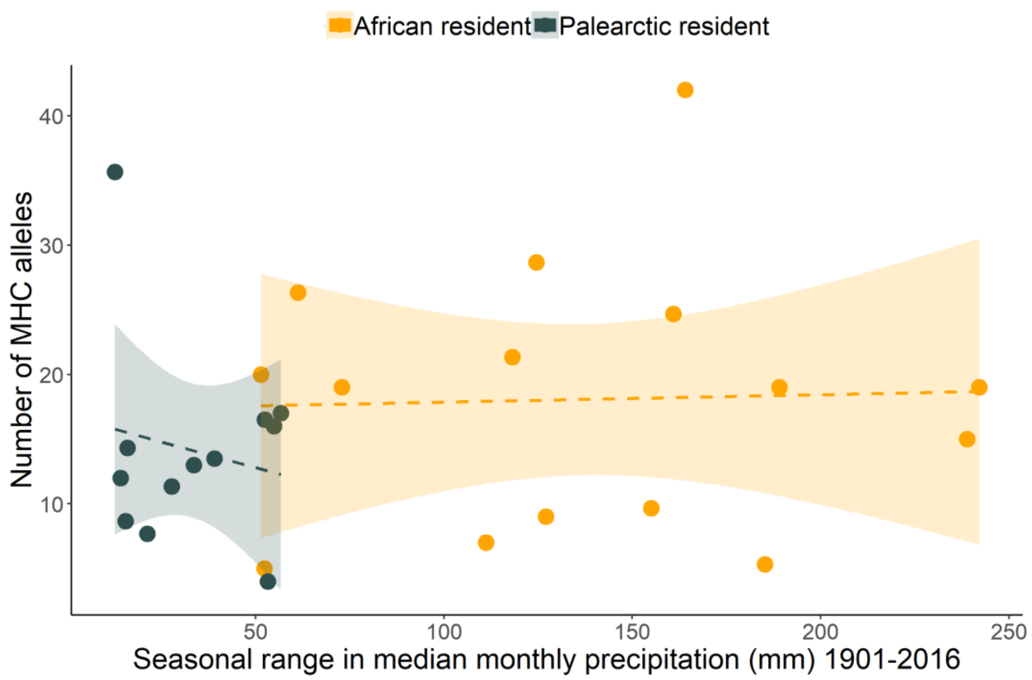

Figure S8 Relationship between the magnitude of the seasonal range of precipitation and the mean number of MHC-I alleles per individual in African and Palearctic resident species ($n_{species}$ = 27). Seasonal range = mean precipitation of the wettest month – mean precipitation of the driest month (wettest and driest months determined from the median precipitation per month from 1901 to 2017). Dashed lines and shaded areas show linear regressions with 95% confidence intervals. The African resident species experience a wider range of monthly precipitation across the year compared to Palearctic species. However, the number of MHC-I alleles each species has does not appear to be influenced by the magnitude of the seasonal range of precipitations experienced.

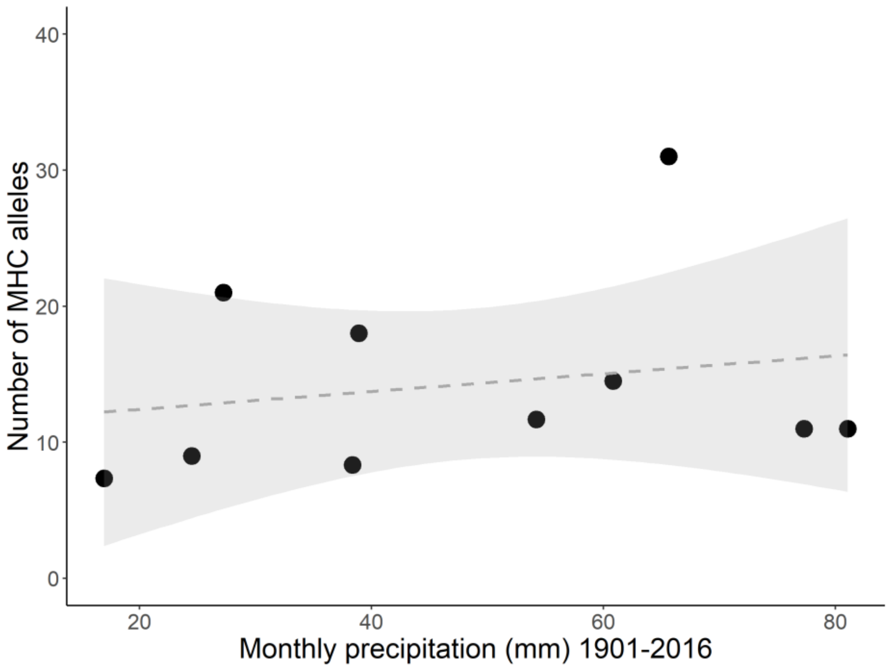

Figure S9 Relationship between the number of MHC-I alleles and precipitation in migratory species ($n_{species}$ = 10). Median precipitation calculated from the combined precipitation data between May to July in the Palearctic and November to January in Africa. Dashed line and shaded area show linear regressions with 95% confidence intervals

APPENDIX S10: INVESTIGATION OF WHY A RELATIONSHIP BETWEEN NUMBER OF MHC-I ALLELES AND PRECIPITATION WAS NOT OBSERVED IN THE MIGRATORY SPECIES

S10.1 Is the difference between migrant and African resident species explained by sample size?

The smaller sample size of migratory species in our dataset compared to residents may have led to insufficient power to detect the relationship we observed in the African resident species. However, this does not appear to be the case because when randomly sub-sampling the same number of species (10) from the African residents 10 times we recovered the same strong relationship between precipitation and number of MHC-I alleles (Table S12). Therefore, if a similar relationship existed in the migratory species, a sample size of 10 should have been sufficient to detect it.

S10.2 Is the difference between migrant and African resident species explained by occupying different regions?

The absence of a relationship between number of MHC-I alleles and precipitation in the migratory species could be due to migrants occupying different regions to residents. To test this we calculated the area of overlap in the distribution ranges of all the species used in this study. Before conducting these calculations, we first transformed the projection of the shapefiles to a Lambert azimuthal equal-area projection to ensure equal dimensions of each cell regardless of distance from the poles. Next, we used the 'intersect' function of the R package 'raster' (1) to create shapefiles of the overlap for each pairwise species comparison. We then used the 'area' function, again within the 'raster' package, to calculate the area of

each of these overlap shapefiles as well as the full area of each species' distribution and calculated the percentage of overlap between each migrant and resident species (2162 pairwise species comparisons: Table S13). The ranges of migrants overlapped by at least 25% in their breeding grounds with six or more Palearctic species and in their wintering grounds with four or more African resident species (Fig. S10.2). The median percentage of overlap between the seasonal ranges of the migratory species and the regional residents is 40.4% for the breeding ranges and 24.4% for the wintering ranges. Thus the migrants appear to occupy similar regions to those of resident species (Fig. 1).

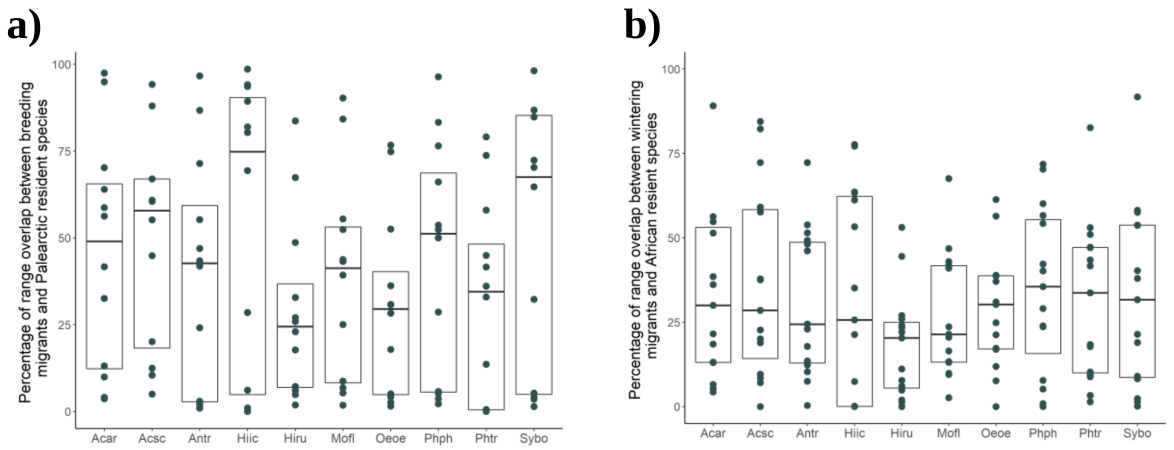

Figure S10.2 Percentage of overlap between the seasonal ranges of migratory species with the Palearctic (**a**) and African (**b**) resident species in the study. Shown using boxplots with all raw data overlaid. Central line of the box depicts the median, the lower and upper hinges correspond to the first and third quartiles. Migratory species codes: Acar = *Acrocephalus arundinaceus*, Acsc = *Acrocephalus scirpaceus*, Antr = *Anthus trivialis*, Hiic = *Hippolais icterina*, Hiru = *Hirundo rustica*, Mofl = *Motacilla flava*, Oeoe = *Oenanthe oenanthe*, Phph = *Phoenicurus phoenicurus*, Phtr = *Phylloscopus trochilus* and Sybo = *Sylvia borin*.

S10.3 Is the difference between migrant and African resident species explained by experiencing different amounts of precipitation?

Another possible explanation for why migrants differ from the resident species in their relationship between MHC-I diversity and precipitation is that they experience different levels of precipitation. This could result in selection on immune genes differing between these groups. We tested whether the precipitation experienced by migratory species during the time they occupy their breeding and wintering ranges differed from that experienced by the resident species during this time. We found no difference in the precipitation experienced by African residents and migratory species during the wintering period (Fig. S10.3, PM = -0.3458, CI = -3.0874 to 2.2869, pMCMC = 0.31, Table S14), but that migrants experienced more precipitation during their breeding period than resident species in the Palearctic (PM = 0.4215, CI = 0.0089 to 0.9004, pMCMC = 0.03).

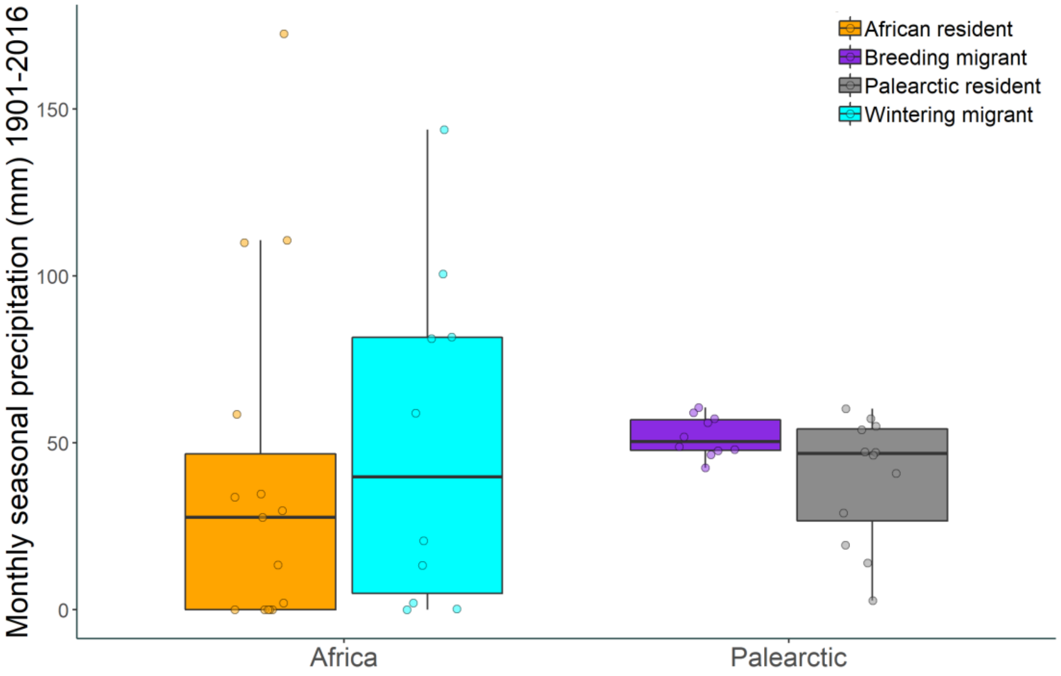

Figure S10.3 Median monthly precipitation experienced by African residents, Palearctic resident and migratory species. Median monthly precipitation calculated for African resident species ($n_{species}$ = 15) and wintering migrants ($n_{species}$ = 10) in Africa between November to January, and for Palearctic resident species ($n_{species}$ = 12) and breeding migrants ($n_{species}$ = 10) in the Palearctic between May to July. Shown in box-plots (central line depicts the median, the lower and upper hinges correspond to the first and third quartiles and whiskers extend to the highest value within 1.5 * the interquartile range) overlaid with the individual data points for each species.

S10.4 Is the difference between migrant and African resident species explained by inaccurate precipitation data for migrants?

It is possible that there are inaccuracies in the distribution maps we used to extract the climate data for the migratory species. All the distribution maps are approximations, which are inherently subject to some degree of error. However, as records of migratory birds when they are in Africa are notoriously lacking, the exact wintering ranges of these species may be subject to a particularly high degree of error (11). However, cross-referencing of the observation records from the citizen-science project eBird (12) against the wintering range maps of the migratory species in our study suggests that these maps are generally accurate (cross-referencing performed on 5th March 2019).

S10.5 Is the difference between migrant and African resident species explained by migrants spending less time in Africa?

Another possible explanation for the migrants differing from African residents in their relationship between MHC-I diversity and precipitation is that they are only in Africa for a restricted period of the year. Thus, it is possible that this is not sufficient to drive selection on

their immune genes. We tested this idea by examining whether the relationship between MHC-I diversity and precipitation differed in the African resident species when we only considered the precipitation they experience during the time the migrants are present in Africa (November to January). When we examined the data this way, we no longer observed the strong association between number of MHC-I alleles and precipitation in the African resident species (Fig. 4, PM = 0.0010, CI = -0.0053 to 0.0082, pMCMC = 0.30, Table S15). This suggests that the period the migrants experience precipitation in Africa may not be sufficient to mediate selection on immune genes. However, sub-setting the data in this way for the African residents is complicated by the different seasonal patterns of precipitation across Africa (Fig. S10.5). Depending on where in Africa species live, the period from November to January can be either dry or wet. This dichotomy in the data may cloud the relationship between number of MHC-I alleles and precipitation, given that these conditions are not necessarily representative of the precipitation received throughout the rest of the year (Fig. S10.5). To address this, we examined the relationship in the African resident species between number of MHC-I alleles and precipitation during the three wettest and the three driest months for each species (i.e. the exact months differ depending on region in which each species lives). Although there was a trend for a positive association between precipitation during both the wettest and driest periods and number of MHC-I alleles (wettest months: PM = 0.0048, CI = -0.0019 to 0.0124, pMCMC = 0.10; driest months: PM = 0.0104, CI = -0.0085 to 0.0355, pMCMC = 0.10, Table S15), it was only when combining the data across the three wettest and three driest months that we retrieved the strong association between precipitation and number of MHC-I alleles in the African resident species (PM = 0.0176, CI = 0.0041 to 0.0300, pMCMC <0.001, Table S15). However, analysing the data using the precipitation of the remaining six months (i.e. with intermediate precipitation) also resulted in a similar association between precipitation and number of MHC-I alleles (PM =

0.0157, CI = 0.0043 to 0.0285, pMCMC <0.001, Table S15), suggesting that it is not one particular period that drives this relationship, but rather the cumulative effect of precipitation. It is likely that the longer the migratory species are in Africa, the greater the diversity of pathogens they are exposed to in the wetter regions.

It should be noted that we chose November to January to represent a period when all ten of the migratory species are likely to be in Africa, given variation between the species in arrival and leaving dates (13,14,23,24,15–22). However, the full duration of the wintering period is generally longer than three months, meaning that the migratory species are likely to also experience the precipitation in Africa over a more extended period. To check whether this influenced our results, we tested whether the relationship between number of MHC-I alleles and precipitation differed in migratory species when we analysed precipitation data from their African wintering ranges between October and March and their Palearctic breeding ranges between April and September. However, there was still no evidence of a relationship between number of MHC-I alleles and precipitation in either the breeding or wintering ranges of the migratory species (breeding range: PM = -0.0148, CI = 0.0318 to 0.3800, pMCMC = 0.38; wintering range PM = -0.0016, CI = -0.0080 to 0.0046, pMCMC = 0.34, Table S16). This suggests that the lack of relationship was robust to variation in the exact months designated as breeding or wintering for the migrants.

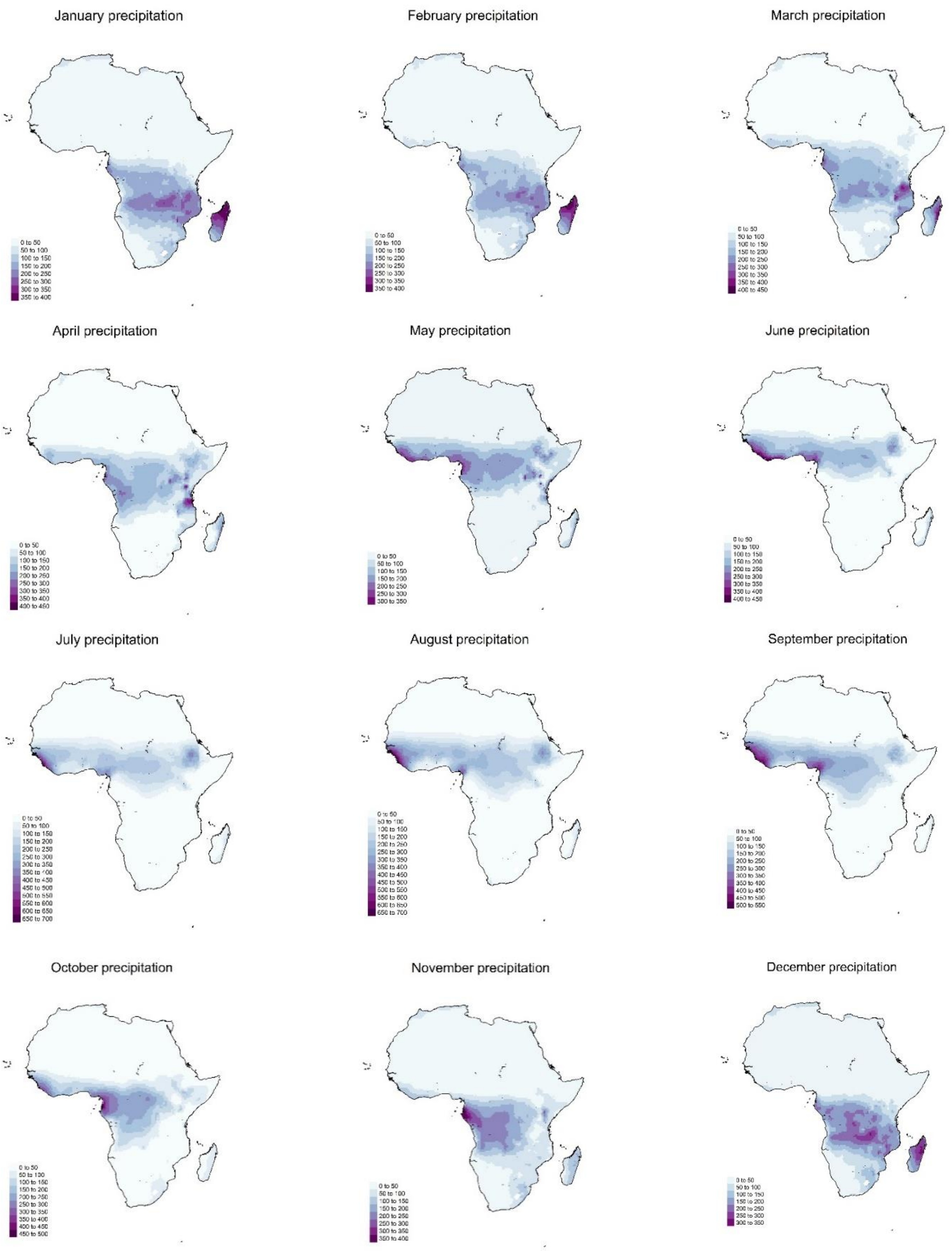

Figure S10.5 Median precipitation (mm) each month across Africa from 1901 to 2017. Precipitation data based on monthly records from 1901 to 2017 collated by the Climatic Research Unit at the University of East Anglia.

References

1. Hijmans RJ, van Etten J, Cheng J, Mattiuzzi M, Sumner M, Greenberg JA, et al. R Package ‘raster ’: geographic data analysis and modeling. 2019.

2. Moreau RE. The Immigrant Palaearctic bird fauna. In: The Bird Faunas of Africa and Its Islands. London, UK: Academic Press; 1966.

3. Hartl DN, Clark AG. Principles of Population Genetics. 4th ed. Sunderland, MA: Sinauer Associates; 1997.

4. Carnaval AC, Hickerson MJ, Haddad CFB, Rodrigues MT, Moritz C. Stability predicts genetic diversity in the brazilian Atlantic forest hotspot. Science. 2009;323:785–90.

5. Tamura K, Dudley J, Nei M, Kumar S. MEGA Version 4. Mol Biol. 2007;24(8):1596–9.

6. O’Connor EA, Cornwallis CK, Hasselquist D, Nilsson JÅ, Westerdahl H. The evolution of immunity in relation to colonization and migration. Nat Ecol Evol. 2018;2(5):841–9.

7. Gaston KJ. Range Size. In: Harvey PH, May RM, editors. The Structure and Dynamics of Geographic Ranges. Oxford: Oxford University Press; 2003.

8. Bock CE, Ricklefs RE. Range size and local abundance of some North American songbirds : A positive correlation. Am Nat. 2012;122(2):295–9.

9. Brown JH. The Abundance and Distribution of Species. In: Macroecology. Chicago and London: The University of Chicago Press; 1995.

10. Gutiérrez JS, Piersma T, Thieltges DW. Micro- and macroparasite species richness in birds: the role of host life history and ecology. J Anim Ecol. 2019;88(8):1226–39.

11. Walther BA, Rahbek C. Where do Palearctic migratory birds overwinter in Africa ? Dansk Ornitol Foren Tidsskr. 2000;96:4–8.

12. Sullivan BL, Aycrigg JL, Barry JH, Bonney RE, Bruns N, Cooper CB, et al. The eBird

enterprise: An integrated approach to development and application of citizen science. Biol Conserv. 2014;169:31–40.

13. Cramp S. Handbook of the Birds of Europe, the Middle East and North Africa: The Birds of the Western Palearctic Volume V: Tyrant Flycatchers to Thrushes. 1988.

14. Willemoes MK, Tøttrup AP, Thorup K. Migration of the Common Redstart (*Phoenicurus phoenicurus*). Auk. 2013;130(2):258–64.

15. Thorup K, Vardanis Y, Tøttrup AP, Kristensen MW, Alerstam T. Timing of songbird migration: Individual consistency within and between seasons. J Avian Biol. 2013;44(5):486–94.

16. Saino N, Rubolini D, Jonzén N, Ergon T, Montemaggiori A, Stenseth NC, et al. Temperature and rainfall anomalies in Africa predict timing of spring migration in trans-Saharan migratory birds. Clim Res. 2007;35:123–34.

17. Liechti F, Scandolara C, Rubolini D, Ambrosini R, Korner-Nievergelt F, Hahn S, et al. Timing of migration and residence areas during the non-breeding period of barn swallows Hirundo rustica in relation to sex and population. J Avian Biol. 2015;46(3):254–65.

18. Arlt D, Olsson P, Fox JW, Low M, Pärt T. Prolonged stopover duration characterises migration strategy and constraints of a long-distance migrant songbird. Anim Migr. 2015;2(1):47–62.

19. Salewski V, Bairlein F, Leisler B. Different wintering strategies of two Palearctic migrants in West Africa - A consequence of foraging strategies? Ibis. 2002;144(1):85–93.

20. Horns JJ, Buechley E, Chynoweth M, Aktay L, Çoban E, Kırpık MA, et al. Geolocator tracking of Great Reed-Warblers (*Acrocephalus arundinaceus*) identifies key regions for migratory wetland specialists in the Middle East and sub-Saharan East Africa. Condor. 2016;118(4):835–49.

21. Lemke HW, Tarka M, Klaassen RHG, Åkesson M, Bensch S, Hasselquist D, et al. Annual

cycle and migration strategies of a trans-Saharan migratory songbird: A geolocator study in the Great Reed Warbler. PLoS One. 2013;8(10):1–10.

22. Arizaga J, Willemoes M, Unamuno E, Unamuno JM, Thorup K. Following year-round movements in Barn Swallows using geolocators: Could breeding pairs remain together during the winter? Bird Study. 2015;62(1):141–5.

23. Schmaljohann H, Buchmann M, Fox JW, Bairlein F. Tracking migration routes and the annual cycle of a trans-Sahara songbird migrant. Behav Ecol Sociobiol. 2012;66(6):915–22.

24. Lerche-Jørgensen M, Willemoes M, Tøttrup AP, Snell KRS, Thorup K. No apparent gain from continuing migration for more than 3000 kilometres: Willow warblers breeding in Denmark winter across the entire northern Savannah as revealed by geolocators. Mov Ecol. 2017;5(1):1–7.
